# Exploring the role of T helper subgroups and their cytokines in the development of pregnancy-induced hypertension

**DOI:** 10.3389/fimmu.2023.1126784

**Published:** 2023-06-05

**Authors:** Qianqian Zhou, Youcheng Wu, Dongmei Zhang

**Affiliations:** Tongren Hospital, Shanghai Jiao Tong University School of Medicine, Shanghai, China

**Keywords:** pregnancy-induced hypertension (PIH), Th1 cell, Th2 cell, cytokines, immune

## Introduction

According to the modern tenets of reproductive immunology, pregnancy can be regarded as a triumphant outcome of a natural allograft. The fetus successfully evades rejection owing to the presence of the immune barricade of the placenta, immunosuppressive cells, and immunomodulators within the maternal environment. Thus, a successful pregnancy depends on the balance of immunity between the fetus and the mother. In other words, maternal-fetal tolerance is the key to a successful pregnancy. Furthermore, the interaction between the maternal and fetal systems, as well as the impact of fetal cells circulating within the maternal bloodstream and inducing an inflammatory response, warrants consideration (1). Loss of maternal immune tolerance, causing immune rejection, may lead to pregnancy pathology such as pregnancy-induced hypertension (PIH) (2), abortion, and other conditions (3). PIH is a pregnancy-specific disorder characterized by the onset of hypertension (systolic blood pressure ≥140 mmHg or diastolic blood pressure ≥90 mmHg) after 20 weeks of gestation in the absence of proteinuria or other organ dysfunction. The severity of PIH is classified into three stages: mild, moderate, and severe. PIH is a serious condition that can lead to significant maternal and fetal morbidity and mortality. The risks associated with PIH include placental abruption, fetal growth restriction (FGR), preterm birth, and maternal organ damage, such as liver and kidney dysfunction (4). If left untreated, PIH can progress to preeclampsia (PE) or eclampsia, which are even more severe conditions that can be life-threatening for both mother and fetus (5). Adopting an immunological perspective can be instrumental in uncovering and comprehending the etiology and pathogenesis of PIH, thereby providing valuable insights for the prevention, diagnosis, treatment, and care of PIH.

The most recognized model of PIH is poor placentation due to abnormal spiral artery formation, while immune modulation of trophoblast invasion is thought to play an important role in the pathogenesis of PIH (6). The maternal immune response to fetal alloantigens is dynamic and ideally shifts between immune suppression and response during placentation, with pregnancy gestation, and then birth (7). In a normal pregnancy, the maternal immune system undergoes changes to accommodate the fetus and prevent it from being recognized as foreign (8). This includes the production of certain immune cells and molecules that suppress the immune response and promote tolerance to the fetus. The rejection reaction, on the other hand, occurs when the immune system recognizes the fetus as foreign and launches an attack against it. However, in pregnant women with PIH, there is a decrease in the protective response and an increase in the rejection reaction (9). This means that the mother’s immune system is more likely to perceive the fetus as a threat and attack it, potentially leading to complications. Chemokine gene silencing in decidual stromal cells restricts the entry of T cells into the maternal-fetal interface. Effector T cells cannot accumulate in the decidua, the special stromal tissue that surrounds the fetus and placenta (10). Moreover, the regulation and recruitment of inducible regulatory T cells by trophoblast cells occur during early pregnancy (11). A large number of studies have shown that the T helper (Th) cells subgroup and their secreted cytokines play a core regulatory role in pregnancy immunity and are closely related to the occurrence of PIH (7, 9, 12, 13).Therefore, even in the presence of a small number of T cells, the effects of T cells themselves and their secreted cytokines are noteworthy.

**Table 1:** The association of Th cells and cytokines with PIH.

Cell Type	cytokines	The association with PIH	References
Th1 cell	IL-2	Immune dysregulation	(14)
		Destruction of trophoblast cell	(15)
		Regulates the secretion of VEGF	(16)
	INF-γ	Induces endothelial cell dysfunction and apoptosis	(17, 18)
		Promotes production of ROS	(19)
		Inhibits trophoblast invasion	(20)
		Up-regulates the expression of HLA-G	(21)
		Promotes the development of an inflammatory environment	(22)
	TNF-α	Mediating maternal-fetal immune regulation	(23)
		Affects trophoblast infiltration of maternal SA	(24, 25)
		Activates neutrophils to release elastic proteinase and promote neutrophils	(26)
		Vascular endothelial damage	(27)
		Modulates anticoagulant factors	(28)
		Augments apoptosis of placental trophoblast cells	(29, 30)
		Regulates plasma leptin levels	(31–33)
		Causes the involvement of trophoblast cells and decreased infiltration ability	(34)
		Enhances local cellular immune response	(35)
Th2 cell	IL-6	Facilitates the fusion of maternal and nourishing cells.	(36, 37)
		Participates in the formation of the placental blood vessels	(38, 39)
	IL-4	Maintenance of vascular integrity and endothelial function	(40, 41)
		Modulates the immune response	(42)
		Imbalances in the Th1/Th2 ratio	(43, 44)
Th17	IL-17	Induces the production of other pro-inflammatory cytokines and chemokines	(45)
		Stimulates the production of ROS	(46)
		Promotes the infiltration of neutrophils and macrophages	(47)
	IL-21	Differentiation and activation of Th17 cells	(48)
	IL-22	Regulates angiogenesis and VEGF	(49)
Tregs	TGF-β	Ameliorates PIH	(50)
	IL-10	Immunosuppression	(51, 52)

**Figure 1:**
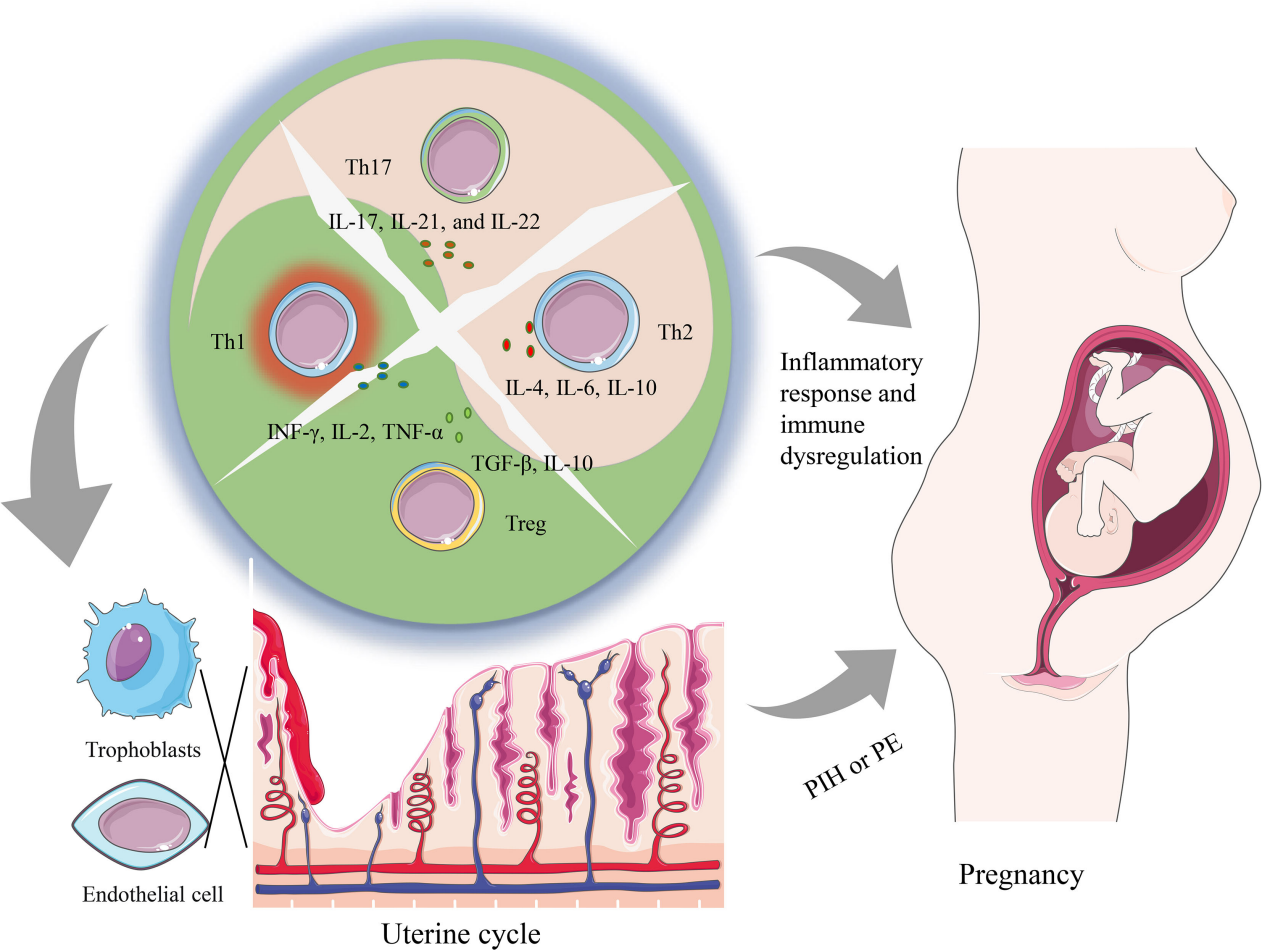
A summary of how imbalanced T helper cell functions can lead to both systemic changes as well as local changes contributing to PIH pathology. The dysregulation of Th cells containing Th1, Th2, Th17, and Tregs subsets alters the cytokine environment, which can promote a local and systemic inflammatory response that is associated with the occurrence of PIH. Furthermore, locally during placentation, an imbalance of T helper cells and related cytokines can hinder the infiltration of trophoblasts and induce the dysfunction of vascular endothelial cells, and further prompt the generation of PE.

T helper subsets primarily achieve their physiological functions through the release of cytokines. These cytokines have broad biological effects, including regulating the activation and proliferation of immune cells, regulating inflammatory responses, and affecting biological processes such as cell proliferation, differentiation, and apoptosis. In this article, we mainly focus on the effects of cytokines produced by T helper subsets on the placental trophoblast and other immune cells, and the association of these effects with the development of PIH (**Figure 1**). In this perspective, we will summarize the role of T helper subsets (TH1/2/17 and Treg) in PIH and explore the role of relevant cytokines in the pathogenesis of PIH.

## Overview of T helper subsets

The Th cell-mediated adaptive immune response represents a critical component in the intricate mechanism of maternal-fetal immune tolerance. Upon encountering a diverse array of cytokine stimuli, the Th0 cells, which originate from initial CD4+ T cells, undergo differentiation into distinct subsets including Th1, Th2, Th17, and Treg cells, each of which assumes discrete biological functions (13). Th1 cells mainly secrete IFN-γ, TNF-α, and other cytokines, which have cytotoxic effects, and can inhibit the invasion of trophoblasts and induce their apoptosis. (53) Th1 cells can also inhibit embryo implantation by enhancing the vitality of decidual macrophages, which is not conducive to the maintenance of pregnancy (54). In contrast to Th1 cells, Th2 cells possess the capacity to produce IL-4 cytokines, which act to promote trophoblast cell proliferation and invasion, enhance uterine receptivity, and confer immune-nutritive and protective benefits to the fetus (55, 56).

Th17 cells mainly mediate inflammatory diseases and autoimmune diseases, and the cytokine IL-17 secreted by Th17 can promote the invasion of extracellular villus trophoblast cells and inhibit their apoptosis (57). Treg cells promote CD4+CD25-T differentiation to CD4+CD25 + Treg by secreting inhibitory cytokines such as TGF-β, showing the advantage of Treg cells at the mother-fetal interface and indirectly playing the role of immunomodulator (58).

Therefore, normal physiological pregnancy mainly presents a Th2-type immune advantage and the Treg cell amplification phenomenon. Th1/Th2 and Th17/Treg balance is an essential condition for maintaining normal pregnancy. On the other hand, Th1/Th2 and Th17/Treg balance play a role in maintaining maternal-fetal immune tolerance (14). Research has reported that the number and function of Th cells and the ratio of Th1/Th2 in patients with PE were significantly decreased (59). Therefore, paying attention to the changes of Th1/Th2 in PIH that may exist before the occurrence of PE is of great significance for understanding the development mechanism of PIH during pregnancy.

## Overview of cytokines secreted from T helper subsets

Cytokines play a complex role in the pathophysiology of PIH and PE (60). The direct effects of cytokines on myocardiocytes to suppress contractility can also have negative consequences for maternal and fetal cardiovascular function (61). The endothelial injury caused by PE can lead to peripheral edema and other complications (62). Overall, the effects of cytokines on the maternal-fetal interface and cardiovascular system in PE are complex and require further research to fully understand. In this article, we will discuss selected cytokines produced by T helper subsets. We will focus on their effects on the immune environment, inflammatory processes, and trophoblast invasion.

## Cytokines secreted from Th1

### IL-2

Notably, IL-2 is a critical cytokine produced by Th1 cells. Studies have demonstrated that Th1 cells are generated from the trophoblast cell and decidual lymphocytes during pregnancy, and their expression is augmented in the placental microenvironment (63). Specifically, studies have shown that the loss or reduction of IL-2 in pregnancy can suppress total natural killer (NK) cell activation, including non-cytolytic NK cells that may play a protective role in the fetal environment (64). Cytotoxic NK cells have been implicated in the development of PE as they are thought to contribute to endothelial dysfunction and inflammation. On the other hand, noncytotoxic NK cells have been shown to play a protective role in maintaining a healthy pregnancy by regulating trophoblast invasion and promoting placental development. Loss or reduction of IL-2 can lead to an increase in cytotoxic NK cells and a decrease in noncytotoxic NK cells, which can contribute to the development of PIH and PE. In addition, IL-2 plays an important role in regulating immune responses and maintaining immune balance, and its abnormal expression may lead to immune dysregulation, promoting the occurrence and development of PIH (14). Hama et al. found that IL-2 played a coordinating role in the destruction of trophoblast cells due to a decrease in the nonclassical human leukocyte antigen-1 (HLA-G) *in vitro* experiments (15). Furthermore, it has been observed that lymphokine-activated killer (LAK) cells derived from decidua are capable of inducing the secretion of vascular endothelial growth factor (VEGF) by nourishing cells (16). Interestingly, heightened IL-2 expression in the decidual milieu has been shown to dampen VEGF release.

### IFN-γ

Interferon-gamma (IFN-γ), a cytokine produced by various immune cells, has been implicated in the pathogenesis of PIH. Studies have shown that PIH patients have elevated levels of IFN-γ, indicating that the dysregulation of IFN-γ may be involved in the pathogenesis of PIH (65). Endothelial dysfunction is a hallmark of PIH, and IFN-γ has been shown to induce endothelial cell dysfunction (17). Specifically, IFN-γ can increase endothelial cell apoptosis, impair endothelial cell proliferation (18), and promote the production of reactive oxygen species (ROS) (19), all of which contribute to the development of PIH. In normal pregnancies, trophoblast cells invade the maternal decidua and remodel the maternal spiral arteries to promote fetal growth (66). However, in PIH, trophoblast invasion is impaired, leading to inadequate placental perfusion and subsequent hypoxia (67). IFN-γ has been shown to inhibit trophoblast invasion by inducing apoptosis of extravillous trophoblast cells and suppressing the expression of invasion-related genes (20). Overexpression of Th1 at the maternal-fetal interface can activate NK cells to up-regulate the expression of HLA-G in the placenta, which is prone to immune rejection, by stimulating the secretion of IFN-γ (21). PIH is associated with an inflammatory response (68), and IFN-γ has been shown to stimulate the secretion of pro-inflammatory cytokines and chemokines (22), promoting the development of an inflammatory environment that contributes to the pathogenesis of PIH. Therefore, the dysregulation of IFN-γ plays a critical role in the pathogenesis of PIH by regulating immune responses, inducing endothelial dysfunction, impairing trophoblast invasion, and promoting an inflammatory response.

### TNF-α

Among several Th1 cytokines, TNF-α is the most closely related to the occurrence of PIH ^(^ 29, 69, 70 ^).^ Under physiological conditions, there is typically a low expression of TNF-α mRNA in the endometrial glandular epithelium, basement membrane, and ovarian stroma of females. However, during gestation, both the developing fetus and decidual tissue are capable of producing TNF-α, which plays a pivotal role in mediating maternal-fetal immune regulation (23). However, TNF-α increased significantly when PIH occurred. Conrad (24) and Cotechini (25) believed that TNF-α could affect trophoblast infiltration of the maternal spiral artery (SA), resulting in blocked angioplasty of SA, stenosis of the vascular cavity, increased resistance, and sensitivity to vasoactive substances. Conrad et al. conducted a study to investigate the association between circulatory inflammatory cytokines and the pathogenesis of PE (24). The study found that the median concentration of plasma TNF-α was twofold higher in women with PE compared to normal third-trimester pregnancy (P < 0.001) and gestational hypertension (P < 0.04). FGR and PE are frequently linked to abnormal maternal inflammation, deficient SA remodeling, and altered uteroplacental perfusion. Cotechini et al. (25) revealed a novel mechanistic association between abnormal maternal inflammation and the development of FGR with features of PE. By administering low-dose lipopolysaccharide (LPS) to pregnant rats during gestational days 13.5-16.5, they demonstrated that abnormal inflammation resulted in FGR mediated by TNF-α. The results indicated that maternal inflammation can cause severe pregnancy complications through a mechanism involving increased maternal levels of TNF-α (25).

At the same time, TNF-α could activate neutrophils to release elastic proteinase and promote neutrophils to adhere to vascular endothelial cells (26), leading to vascular endothelial damage. It can also directly activate vascular endothelial cells, induce the expression of endothelial cell surface adhesion molecules such as vascular cell adhesion molecule-1 (VCAM-1), damage vascular endothelium, and further enhance the activity of neutrophils through the endothelial system (27). TNF-α has the ability to modulate anticoagulant factors (28), thereby promoting a procoagulant state in vascular endothelial cells. Activation of these pathways can potentially contribute to the pathogenesis of PIH. Research has revealed that increased placental synthesis and secretion of TNF-α in patients with PIH can lead to augmented apoptosis of placental trophoblast cells, which in turn impairs their capacity to invade the decidua and spiral arteries, resulting in shallow placental implantation (29). This pathological process can cause a restructuring of the uterine spiral artery architecture, leading to placental ischemia, hypoxia, and metabolic disturbances (30). In recent years, it has been found that TNF-α can also regulate plasma leptin levels in PIH (31). High levels of TNF-α and leptin may act on trophoblast cells and vascular endothelial cells together to impair their functions and lead to the occurrence of PIH (32). Leptin can induce oxidative stress and inflammation in endothelial cells, leading to endothelial dysfunction and injury (33). Leptin can also impair trophoblast invasion by inhibiting the expression of adhesion molecules and enzymes required for trophoblast invasion, such as integrins and matrix metalloproteinases (71). Additionally, leptin can induce the production of pro-inflammatory cytokines and chemokines, which further impair trophoblast invasion (72).

The upregulation of TNF-α and other cytokines causes the involvement of trophoblast cells and decreased infiltration ability (34), shallow placental implantation resulting in placental ischemia and hypoxia, enhanced local cellular immune response (35), activation of white blood cells in the villus space leading to vascular endothelial injury, and eventually the occurrence of PIH (73). Evidently, immunological factors play a crucial role in the pathogenesis of shallow placental implantation, vascular endothelial injury, and other related factors in PIH. In the future, continued investigation of molecular immunology is anticipated to elucidate the underlying mechanisms of TNF-α in the context of PIH. The observed increase in Th1 cytokines in PIH patients is likely a consequence of the pathology, rather than a cause. Systemic inflammation resulting from tissue injury can lead to an upregulation of inflammatory cytokines, including TNF-α, IL-2, IFN-γ, and IL-4. These cytokines can then contribute to the pathogenesis of PIH through various mechanisms, such as impairing trophoblast invasion and causing endothelial dysfunction.

## Cytokines secreted from Th2

Cytokines secreted by Th2, such as IL-4, IL-6, and IL-10, can inhibit the Th1 immune response and the activation of NK cells to protect the fetus (74). These cytokines mainly participate in B cell proliferation and maturation, which can increase the antibody-mediated immune response. 

### IL-6

IL-6 can stimulate B cells to produce antibodies to stimulate the proliferation and differentiation of cytotoxic T lymphocytes (CTL) (75). Both the placenta and decidua in early pregnancy contain IL-6 mRNA, suggesting that IL-6 may work in conjunction with other factors to facilitate the fusion of maternal and nourishing cells (36, 37). IL-6 also participates in the formation of the placental blood vessels. IL-6 can promote the proliferation and migration of endothelial cells, and stimulate the release of angiogenic factors, such as vascular endothelial growth factor (VEGF), which further promote angiogenesis (38). Excessive IL-6 in late pregnancy is involved in the pathological process of PIH (39).

### IL-4

IL-4 is also an important cytokine involved in immune regulation and inflammation and has been found to be decreased in the serum and placenta of women with PE compared to those with normal pregnancy, suggesting that it may be involved in the pathogenesis of the disease (68, 76). IL-4 is known to play a role in the maintenance of vascular integrity and endothelial function, which are key factors in the development of PIH (40). Reduced IL-4 levels could therefore result in impaired eNOS activity and decreased NO-mediated vasodilation, contributing to hypertension and other cardiovascular complications in pregnancy (41). Furthermore, IL-4 may also modulate the immune response and contribute to the development of PIH through its effects on T-helper cell differentiation and cytokine production (42). Specifically, decreased IL-4 levels have been associated with an imbalance in the Th1/Th2 ratio, which may contribute to the pro-inflammatory state seen in PIH (43). It can be concluded that IL-4 is involved in the regulation of vascular function, immune response, and inflammation, which are important implications in the pathogenesis of PIH.

In order to deeply understand the relationship between Th cell subsets and their differentiated cytokines and PIH, Saito et al. (44) investigated Th1 and Th2 cytokines secreted by peripheral blood mononuclear cells (PBMC) of patients with hypertensive diseases during pregnancy by enzyme-linked immunosorbent assay (ELISA). The results showed that the level of Th1 cytokines secreted by PBMC in PIH patients was significantly higher than that in the normal control group, and the ratios of TNF-α/IL-4, IL-2/IL-4, and IFN-γ/IL-4 were also significantly higher. Moreover, the concentrations of the three Th1 cytokines were positively correlated with patients’ MAP. Systemic inflammation resulting from tissue injury can lead to an upregulation of inflammatory cytokines, including TNF-α, IL-2, IFN-γ, and IL-4. Abnormal secretion of these cytokines can then contribute to the pathogenesis of PIH through various mechanisms, such as impairing trophoblast invasion and causing endothelial dysfunction.

A further study showed that compared with normal pregnant women, the ratio of Th1 and Th2 cells increased and the content of Th2 cells decreased in patients with PIH during late pregnancy. The results of this study are consistent with Saito’s report (77). The reciprocal regulation between Th1 and Th2 cells plays a pivotal role in the maintenance of immune homeostasis, particularly in the context of transplant immunology (78). Therefore, aberrant maternal immune responses may serve as a trigger for the onset of PIH (9). Recent investigations have demonstrated that the Th1/Th2 cell ratio in patients with PIH exhibits a tendency towards heightened Th1 activity (79).

## Cytokines from Th17

Th17 cells secrete a number of pro-inflammatory cytokines, including IL-17, IL-21, and IL-22, which have been found to be elevated in the circulation of women with PIH (80). These cytokines may contribute to the development of hypertension and endothelial dysfunction, which are key features of PIH.

## IL-17

IL-17, in particular, has been implicated in the pathogenesis of PIH (81). It induces the production of other pro-inflammatory cytokines and chemokines, such as IL-6 and TNF-α (45), which contribute to the development of hypertension and endothelial dysfunction. IL-17 also stimulates the production of reactive oxygen species (ROS) (46), which can lead to oxidative stress and endothelial damage. Additionally, IL-17 promotes the infiltration of neutrophils and macrophages into the placenta (47), which can further contribute to inflammation and tissue damage.

## IL-21 and IL-22

IL-21 and IL-22 have also been shown to be elevated in women with PIH. IL-21 promotes the differentiation and activation of Th17 cells, and can enhance the production of IL-17 and other pro-inflammatory cytokines (48). IL-22, on the other hand, has been implicated in the regulation of angiogenesis and VEGF signaling (49), which are important processes in the development of placental vascularization and function.

Taken together, the cytokines produced by Th17 cells are important contributors to the pathogenesis of PIH, promoting inflammation, oxidative stress, endothelial dysfunction, and vascular damage. Targeting these cytokines may provide a potential therapeutic strategy for the prevention and treatment of PIH.

## Cytokines from Tregs

Tregs are a subset of T cells that regulate immune responses and maintain tolerance to self-antigens. They produce cytokines such as IL-10 and TGF-β, which have anti-inflammatory and immunosuppressive effects. Several studies have reported lower levels of Tregs in women with PIH compared to normotensive pregnant women (82). This suggests that a deficiency in Tregs may contribute to the development of PIH. In addition, decreased production of IL-10 and TGF-β has been observed in women with PIH (83), further supporting the role of Tregs in the development of PIH. Moreover, it has been shown that administration of TGF-β can ameliorate PIH in a rat animal models study (50). This suggests that Tregs and their cytokines may have therapeutic potential for the treatment of PIH.

### IL-10

IL-10 is a kind of cytokine that has a variety of biological activities and its most important role is in immunosuppression. Many researchers have found that IL-10 can inhibit the expression of TNF-α, INF-γ, major histocompatibility complex (MHC-II) molecules, and B7 adhesion molecules on phagocytes, and block the killing effect of NK (51). The relative lack of IL-10 will increase the content of immune factors, resulting in the breakdown of the Th1/Th2 balance, resulting in the enhancement of the Th1 immune response, leading to the occurrence of PIH (52).

## Discussion and conclusion

In brief, the etiology and pathogenesis of PIH in immunological investigations are posited to arise from the dysregulation of maternal-fetal immune homeostasis or immune tolerance, characterized by diminished Th2-mediated immunosuppression and/or heightened Th1-mediated cellular immune activation **Table 1**.

PIH represents a prevalent condition in obstetrics and has been a subject of intense research. Numerous etiological and pathogenic mechanisms have been proposed, including the immune response theory, oxidative stress theory, capillary endothelial injury theory, and others. However, thus far, none of these theories have fully accounted for the multifaceted pathophysiology of PIH. Here, we briefly summarized the close relationship between Th1 cells, Th2 cells, and the released cytokines and PIH from the perspective of immunology, and discussed the possible occurrence and development mechanism. Overall, although PIH is caused by many factors, the immune factor plays a pivotal role. Therefore, it may be more effective to prevent, diagnose, treat, and care for PIH by focusing on immunological indicators.

The pathogenesis of PIH is still unclear, which makes clinical diagnosis and treatment difficult. The current diagnosis of PIH and PE mainly focuses on hypertension, proteinuria, serum biochemical abnormalities, and fetal growth. It can be seen from this review that the pathogenesis of PIH may be related to immune factors to a certain extent. In recent years, researchers have suggested that the serum levels of inflammatory factors associated with the pathogenesis of PIH and PE may become part of the diagnostic criteria. For example, a study by Li et al. found that serum levels of IL-6 and TNF-α were significantly higher in women with early-onset PIH compared to normotensive pregnant women (29). They proposed that serum levels of IL-6 and TNF-α could be used as potential biomarkers for early diagnosis of PIH.

Significantly, immune modulation is anticipated to emerge as a novel therapeutic target for PIH and PE in clinical management and holds considerable promise in ameliorating maternal mortality. This approach aims to modulate the immune response by regulating key immune pathways and cytokines, such as interleukin-6 (IL-6) and interleukin-10 (IL-10), and may improve outcomes for women with PIH and PE. Immune modulation strategies for the treatment of PIH and PE include the use of immunomodulatory agents and the development of novel targeted therapies. For example, Tinsley et al. (84) used a PIH rat model of deoxycorticosterone acetate (DOCA)/salt-low renin, which exhibits features of hypertension, proteinuria, endothelial dysfunction, and intrauterine growth restriction (IUGR). Furthermore, suppression of the immune system with either azathioprine (Aza) or mycophenolate mofetil (MMF) during the second half of pregnancy significantly reduced hypertension, proteinuria, and endothelial dysfunction, as well as increased the proinflammatory Th1 cytokine profile in rats treated with DOCA/salt, which alleviated the development of PIH. Medications that target the immune system, such as anti-inflammatory drugs or targeted immunotherapy agents, may be beneficial for PIH patients with an overactive immune system. However, medication interventions should be tailored to the individual patient based on their immunological profile and other medical conditions.

## Author contributions

QZ, YW, and DZ wrote the first draft of the manuscript. All authors contributed to the article and approved the submitted version.
